# Bone mesenchymal stem cell therapy for ovariectomized osteoporotic rats: a systematic review and meta-analysis

**DOI:** 10.1186/s12891-019-2851-2

**Published:** 2019-11-20

**Authors:** Zhenxiong Jin, Jinman Chen, Bing Shu, Yanhua Xiao, Dezhi Tang

**Affiliations:** 1 Longhua Hospital, Shanghai University of Traditional Chinese Medicine, Shanghai, 200032 China; 2 Institute of Spine, Shanghai University of Traditional Chinese Medicine, Shanghai, 200032 China; 3 Key Laboratory of Theory and Therapy of Muscles and Bones, Ministry of Education, Shanghai, 200032 China; 4 Jinggangshan University, Ji’an, 343009 China

**Keywords:** Bone mesenchymal stem cells, Fracture, Meta-analysis, Osteoporotic, Ovariectomy

## Abstract

**Background:**

Previous studies have found that bone mesenchymal stem cells (BMSCs) were capable of self-replication, multi-differentiation, and regeneration. The aim of this study was to carry out a systematic review and meta-analysis of the efficacy of BMSC therapy for ovariectomized rats.

**Methods:**

The PubMed, Embase, Web of Science, China National Knowledge Infrastructure, VIP, and Chinese Sinomed databases were searched systematically from their initiation date to October 5, 2018. Two researchers independently screened the literatures, which used the bone mineral density (BMD), total bone volume by total tissue volume (BV/TV) (%), and trabecular thickness/spacing (Tb/Sp) as the outcome measures.

**Results:**

Five eligible studies were selected. In the BMSC treatment groups, the BMD values and normalized BV/TV values remarkably increased. In addition, in the BMSCs plus other treatment groups, the BMD and Tb/Sp values significantly increased.

**Conclusion:**

This study showed that BMSCs could accelerate callus maturity, ossification and restore mechanical properties of bones in osteoporotic fractures.

## Background

Osteoporosis is a systemic skeletal disease characterized by low bone mass and degradation of the bone microstructure, with consequent increases in the fragility of bone and risk of fracture [1]. A large number of complications have been discovered, which seriously threaten people’s lives and health. Osteoporotic fracture is a serious complication of osteoporosis. Osteoporotic fractures occur following minimal violence or, in some cases, without trauma [2]. In the United States, about 9.9 million people suffer from osteoporosis and 43.1 million have a low bone mineral density (BMD) with an increased likelihood of fractures [1]. Moreover, in China, about 112 million people suffer from osteoporosis, and the prevalence of fractures in people aged more than 50 years of age is 26.6%, with nearly one third of them due to osteoporosis [3].

Tissue engineering technology has been rapidly developed in the fields of bone and cartilage tissue construction, blood vessels, nerves, skin, and so on. As an important part of tissue engineering technology, stem cells have received extensive attention owing to their unique advantages. Bone marrow mesenchymal stem cells (BMSCs) represent a stem cell population that can be harvested from the bone marrow [4] and can differentiate into osteoblasts, fat, cartilage, neuron, and so on. Moreover, BMSCs have attracted much attention because of their advantages, such as easy material extraction and self-renewal [5]. Furthermore, the advantages of autologous transplantation with BMSCs include small trauma, no rejection, and few post-transplant complications [6]. It can effectively avoid bone defects and healing delay in traditional autologous bone transplants. Hence, BMSCs are widely used in cell-level clinical studies on bone and cartilage tissue. But at present, most of the treatment methods of osteoporotic fracture include mainly surgery and conservative intervention, and the severity of osteoporosis affects the occurrence, development, and prognosis of osteoporotic fractures [2]. Additionally, the perioperative treatment of osteoporotic fractures requires attention to prevent the occurrence of re-fracture [7]. Therefore, with the increase of age, the decline of physical health or other factors, new progress in innovative therapy is needed, so cell-based repair therapy has become a promising therapeutic strategy [8].

However, only a small number of the reports have been reported on the clinical application of BMSCs [9], and it is the treatment of osteoporosis, rather than osteoporotic fractures. Most of the stem cell therapy for osteoporotic fractures remains in basic research. For this we focus our attention on animal models. Ovariectomy (OVX) results in the decrease level of estrogen and is well established in investigations of osteoporotic therapies [10]. OVX induces bone loss in animals, and postmenopausal bone loss has many similar features [11], including a rapid decrease in the trabecular bone mass and an increase in bone resorption, and similar skeletal response to therapy with estrogen, bisphosphonates, tamoxifen, calcitonin. These wide-ranging similarities make ovariectomized animal models have been widely used as clinically relevant models of postmenopausal bone loss in women [12]. The ovariectomized (OVX) rat model was approved by the US Food and Drug Administration (FDA) as a preclinical model [13].

Mesenchymal stem cells have been reported beneficial to animal models of OVX [14–19]. However, in most cases, functional improvement occurs despite minimal engraftment at the site of injury, suggesting that BMSCs may have paracrine effects by secreting factors that promote regeneration without attachment [20, 21]. Although BMSC has been reported to be studied on animals, such as rats, mice, and horses. But BMSC has less research articles on animals other than rats. Therefore, the present study aimed to conduct a systematic review and meta-analysis of the efficacy of BMSCs for OVX rats. The findings can contribute to the clinical trials and treatments in the future.

## Methods

### Literature search strategy

Six databases, including PubMed, Embase, Web of Science, China National Knowledge Infrastructure (CNKI), VIP, and Chinese Sinomed, were systematically searched from their inception dates to October 5, 2018. The following keywords were used for the search: (Fracture AND Osteoporosis) OR (Osteoporotic Fracture) AND (Stem Cell), regardless of the language and publication date.

### Data extraction and quality assessment

The studies were selected independently by two reviewers (Jin ZX and Chen JM) by screening the abstracts and full-texts according to the eligibility criteria. During the process, disagreements were resolved by consensus with a third author (Tang DZ). The studies that satisfied the inclusion and exclusion criteria were enrolled in the meta-analysis.

### Eligibility criteria

#### Types of studies

Controlled studies estimating the effects of BMSCs on ovariectomized rats by in vivo administration were searched. The clinical case reports and studies having only in vitro experiments were excluded.

#### Types of participants

To generate osteoporotic rats, the Sprague-Dawley female rats of any age were subjected to bilateral OVX or sham operation (sham).

#### Types of intervention

Any type of BMSC intervention compared with placebo control was included. Placebo control included PBS, PLGA/Col microspheres, 214S, and no treatment.

#### Types of outcome measures

BMD was considered to be the primary outcome measure for evaluating the anti-osteoporosis efficacy by any anti-osteoporosis therapy in preclinical and clinical studies. Thus, in this systematic review and meta-analysis, each study using BMD as a major result of indicators was considered. Second, the outcome indicators included total bone volume by total tissue volume (BV/TV) (%), trabecular thickness/spacing (Tb/Sp), and so on.

### Exclusion criteria

The exclusion criteria were as follows: (1) The topics were non-primary osteoporosis and new compression fractures. (2) The types of literature were clinical trials, in vitro studies, reviews and case reports, conference articles, systematic reviews, and meta-analysis. (3) The interventions were non-BMSCs, including other stem cells, proprietary Chinese medicines, granules, and ointments. (4) The necessary data were not reported.

### Statistical analysis

All the data review and meta-analysis were performed using the Review Manager 5.3 software provided by the Cochrane Collaboration. The difference between the control group and the intervention group was estimated. Continuous variable data were selected for the standardized mean difference (SMD) analysis. Each effect volume was expressed as a 95% confidence interval (CI). Heterogeneity was observed usingthe*I*^2^ test. *I*^2^ ≤ 50% indicated homogeneity between the studies, which was calculated using the fixed-effects model. *I*^2^ >50% indicated heterogeneity between studies, and a random-effects model was applied for the analysis [11].

## Results

### Selection of studies

**Fig. 1:**
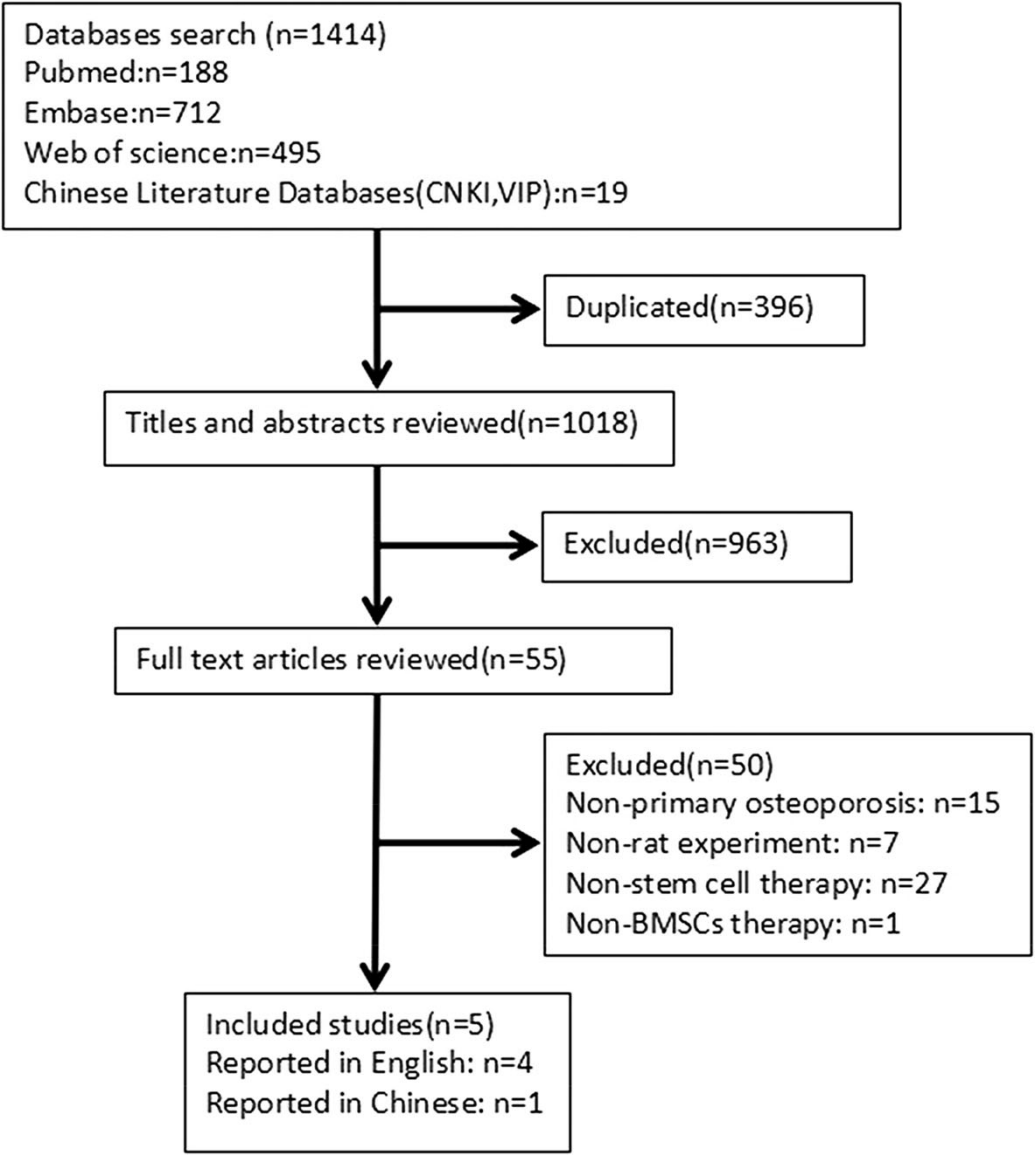
Flow diagram of the literature identification and selection process. A total of 1414 studies were retrieved based on the search strategy described in the Methods section, while 396 of the duplicated ones were excluded. After reviewing the titles and abstracts, 963 records were removed because the studies did not match the eligibility criteria. After reading the full text of the 55 remaining studies, 50 of them were further excluded. Finally, five studies met the inclusion criteria and were selected for this meta-analysis

The detailed flow chart of literature identification and selection process is shown in Fig. 1. A total of 1414 studies were retrieved based on the search strategy described in the Methods section, while 396 of the duplicated ones were excluded. After reviewing the titles and abstracts, 963 records were removed because the studies did not match the eligibility criteria. After reading the full text of the 55 remaining studies, 50 of them were further excluded because the 15 articles topic was non-primary osteoporosis, the 12 articles experimental animal was not the rat and the 10 articles treatment was not based on stem cells, the 12 articles in animal models are non-osteoporotic fractures. Among the remaining six studies, only one study on primary treatment used adipose-derived mesenchymal stem cells (ADSCs) [14]. Therefore, this study could not be included in this meta-analysis. Finally, five studies met the inclusion criteria and were selected for this meta-analysis. One of the included studies was reported in the Chinese language [16], and the remaining studies were reported in English language [15, 17–19].

### Characteristics of the included studies

All the five included studies used female Sprague-Dawley rats, and the number of rats used ranged from 10 to 90. The detailed information of the background diet was not reported in the included studies. All the studies used the ovariectomized animal model. OVX results in a decrease in estrogen produced by ovaries, eventually leading to osteoporosis. Next, the fracture model of the ovariectomized rat models was established. The fracture site was femur in two studies [15, 18], tibia in one study [16], and mandibles in two studies [17, 19]. In the included studies, the outcomes were represented as BMD, BV/TV (%), Tb/Sp, or both or all three. The main features of the five studies are summarized in Table 1.

**Table 1:** Characteristics of studies included in this meta-analysis

Studies	Animals	No. of animals (con/v)	Fracture site	Fracture model	Treatment group	Control group	Treatment duration	Outcomes
Yu, Zr 2012 [15]	SD rats, female, 3-month-old	8/8/8	femur	OVX, fracture	BMSCs, BMSCs+PLGA/COL microspheres	SHAM	3 months	BMD, (BV/TV), (Tb.Sp)
XU ZW 2013 [16]	SD rats, female, (350±8.695)g	18/18/18	tibia	OVX, fracture	BMSCs, BMSCs+Drynaria fortunei extraction	Blank	7/14/28/40 days	BMD
Liu,X 2016 [17]	SD rats, female, (180-220)g	12/12/12	mandibles	OVX, fracture	BMSCs/HA, OPG-BMSCs/HA	HA	4/6/8 weeks	BMD
Li, KC 2016 [18]	SD rats, female, 8-week-old	8/8/6	femur	OVX, fracture	BMSCs, BMSCs+214S	SHAM	2/4 weeks	BMD,(BV/TV),(Tb.Sp)
Xu, RY 2016 [19]	SD rats, female, 3-month-old	5/5	mandibles	OVX, fracture	BMSCs	SHAM	12 weeks	(BV/TV), (Tb.Sp)

Abbreviations: *SD* Sprague-Dawley, *OVX* ovariectomy, *BMD* Bone Mineral Density, *BMSCs* Bone mesenchymal stem cells

### Quality evaluation of the included studies

The quality evaluation [22] of all studies included in this meta-analysis is shown in Table 2. No studies in this meta-analysis specifically described sample-size calculations and allocation concealment, or reported exclusion criteria and outcomes of blinded assessment. Of the five studies, one reported inclusion and exclusion criteria [16], three reported randomization^[15.16.19]^, and two reported potential conflicts of interest and supported funding [15, 19]. Finally, only five published studies met the inclusion criteria. Overall, the quality evaluation of the studies was low.

**Table 2:** Quality evaluation of the included studies

Studies	Sample-size calculation	Inclusion and exclusion	Randomization	Allocation concealment	Reporting animals excluded from analysis	Blinded assessment of outcomes	Reporting potential conflicts of interest and study funding
Yu, ZR 2012 [15]	no	no	yes	no	no	no	yes
XU ZW 2013 [16]	no	yes	yes	no	no	no	no
Liu, X 2016 [17]	no	no	no	no	no	no	no
Li, KC 2016 [18]	no	no	no	no	no	no	no
Xu, RY 2016 [19]	no	no	yes	no	no	no	yes

### Forest map

All five studies used female rats only. The experimental group had a total of 51 rats, and the control group had 49 rats. Depending on the purpose of the study and the method of intervention, two control groups were established: BMSC group and control group, and BMSCs plus other intervention groups and control group. The BMD, BV/TV(%), and Tb/Sp values were compared among the groups. As each study used different time points for data measurement, the last time point was considered for analysis.

### Comparison of BMD value between the BMSC group and the control group

**Fig. 2:**
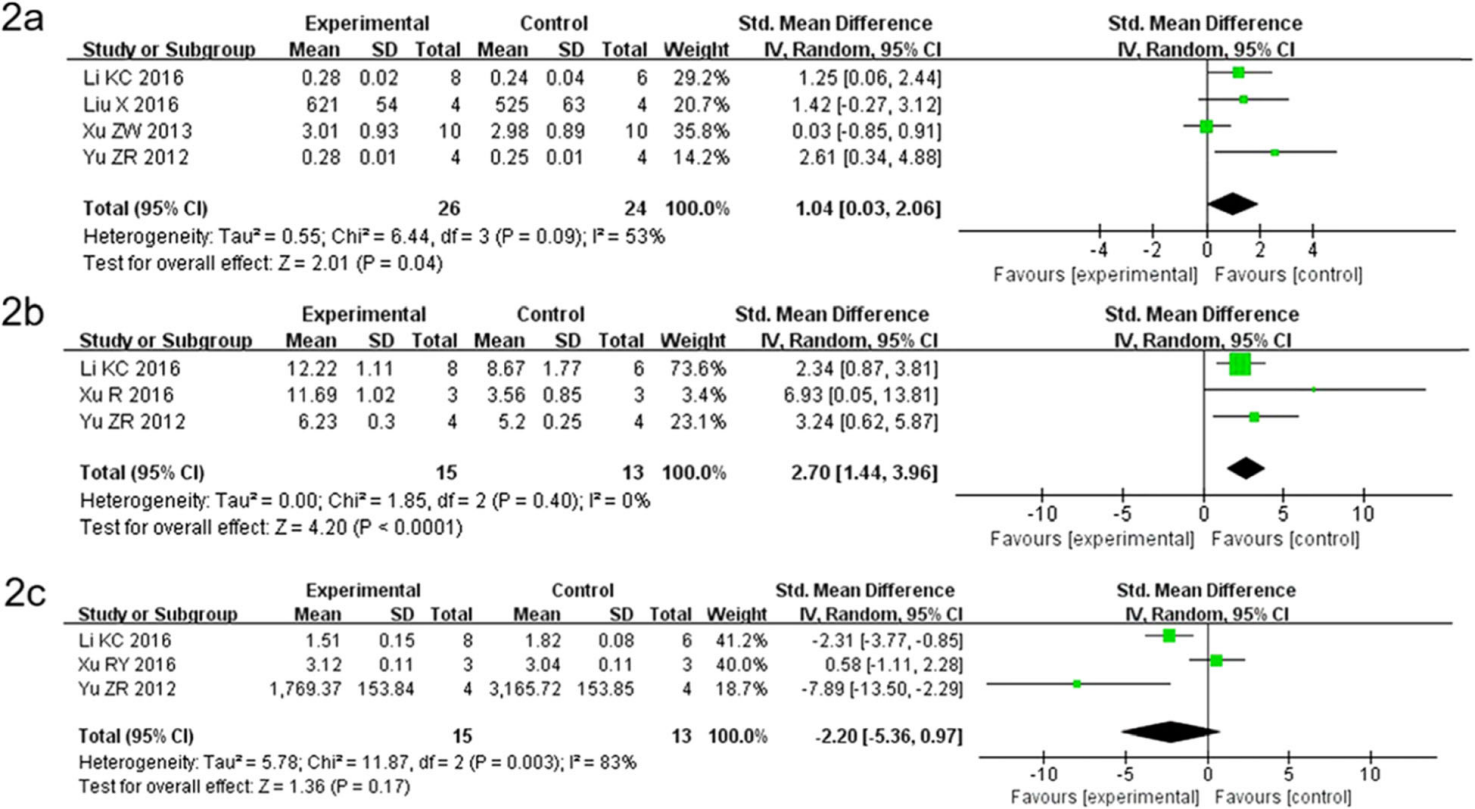
Comparison of BMD, BV/TV (%) and Tb/Sp value between BMSC group and control group. **a** Four studies comparison of BMD value and they were heterogeneous. Therefore, the random-effects model was used for the analysis. The combined effect of SMD was found to be 1.04, with 95% CI = 0.03–2.06 and *P* = 0.04(*P*<0.05). **b** Only three studies comparison of BV/TV(%) value and they were heterogeneous. Hence, the random-effects model was used for the analysis. The combined effect of SMD was found to be 3.90, with 95% CI = 2.32–5.49 and *P*<0.00001. **c** Three studies used the Tb/Sp value as an outcome measure. The combined effect of SMD was found to be 2.20, with 95% CI = 5.36 to 0.97 and *P* = 0.17(*P*>0.05)

Of the five articles, only four used BMD as the primary outcome measure [15–19]. The present meta-analysis involved 26 rats in the experimental group and 24 rats in the control group. The results showed that four studies [15–18] compared the BMSC group with the control group and they were heterogeneous. Therefore, the random-effects model was used for the analysis. The combined effect of SMD was found to be 1.04, with 95% CI = 0.03–2.06 and *P* = 0.04 (Fig. 2a). The diamond lattice did not intersect the invalid line and fell to the right of the invalid line (*P*<0.05). Based on this analysis, the BMD values of one group were statistically significant, indicating that BMSCs increased the BMD in the ovariectomized rats.

### Comparison of BV/TV(%) value between the BMSC group and the control group

Among the five studies, only three studies used the BV/TV (%) value as an outcome measure [15, 18, 19]. The results of this meta-analysis showed that only three studies compared the BMSC group with the control group and they were heterogeneous. Hence, the random-effects model was used for the analysis. The combined effect of SMD was found to be 3.90, with 95% CI = 2.32–5.49 and *P* <0.00001 (Fig. 2b). Based on this analysis, there was a statistically significant difference in BV/TV (%) values between the two groups.

### Comparison of Tb/Sp value between the BMSC group and the control group

Of the five studies, only three studies used the Tb/Sp value as an outcome measure^[15.18.19]^. Increased Tb/Sp suggests increased bone resorption and osteoporosis occurrence. The results of this meta-analysis showed that three studies compared the BMSC group with the control group and they were heterogeneous. Therefore, the random-effects model was used for the analysis. The combined effect of SMD was found to be − 2.20, with 95% CI = − 5.36 to 0.97 and *P* = 0.17 (Fig. 2c). The diamond lattice did intersect the invalid line (*P>*0.05). Based on this analysis, the Tb/Sp values of one group were not statistically significant.

### Comparison of BMD value between BMSCs plus other intervention groups and control group

**Fig. 3:**
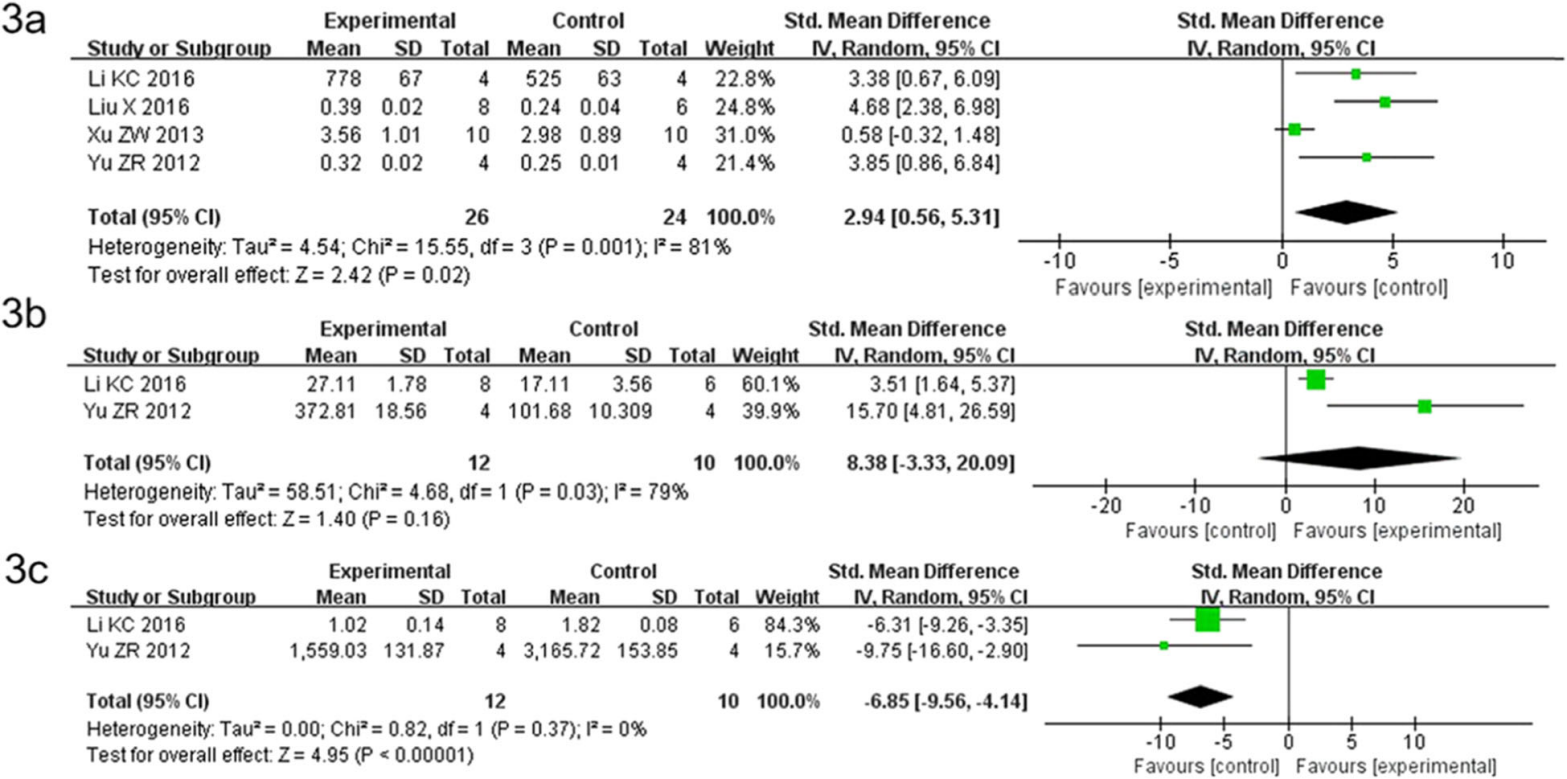
Comparison of BMD, BV/TV (%) and Tb/Sp value between BMSCs plus other intervention groups and control group. The included studies were not limited to a single treatment group. The studies used BMSCs modified by PLGA/Col microspheres, 214S, and traditional Chinese medicine. **a** Comparison of BMD value of this meta-analysis showed that the combined effect of SMD was found to be 2.94, with 95% CI = 0.56–5.31 and *P* = 0.02(*P*<0.05). **b** Among the five articles, two studies used the BV/TV(%) value as an outcome measure. The combined effect of SMD was found to be 8.38, with 95% CI = 3.33 to 20.09 and *P* = 0.17 (*P*>0.05). **c** Only two studies used the Tb/Sp value as an outcome measure. The combined effect of SMD was found to be 6.85, with 95% CI = 9.56 to 4.14 and *P*<0.00001

The included studies were not limited to a single treatment group. The studies used BMSCs modified by PLGA/Col microspheres, 214S, and traditional Chinese medicine. The results of this meta-analysis showed that four studies [15–18] compared the BMSCs plus other intervention groups with the control group and they were heterogeneous. Hence, the random-effects model was used for the analysis. The combined effect of SMD was found to be 2.94, with 95% CI = 0.56–5.31 and *P* = 0.02 (Fig. 3a). The diamond lattice did not intersect the invalid line and fell to the right of the invalid line (*P*<0.05). Based on this analysis, the BMD values in the three groups indicated that BMSCs plus other intervention groups increased the BMD in ovariectomized rats.

### Comparison of BV/TV(%) value between BMSCs plus other intervention groups and control group

Among the five articles, two studies used the BV/TV(%) value as an outcome measure^[15.18]^. The results of this meta-analysis showed that two studies compared the BMSCs plus other intervention groups with the control group and the studies were heterogeneous. Therefore, the random-effects model was used for the analysis. The combined effect of SMD was found to be 8.38, with 95% CI = − 3.33 to 20.09 and *P* = 0.17 (Fig. 3b). The diamond lattice did intersect the invalid line (*P>*0.05). Based on this analysis, the increase in the BV/TV (%) value in ovariectomized rats due to BMSCs injection was not significantly different.

### Comparison of Tb/Sp value between BMSCs plus other intervention groups and control group

Of the five studies, only two studies used the Tb/Sp value as an outcome measure^[15.18]^. Increased Tb/Sp suggests that osteoporosis may occur. The results of this meta-analysis showed that three studies were heterogeneous and compared the BMSCs plus other intervention groups with the control group. Hence, the random-effects model was used for the analysis. The combined effect of SMD was found to be − 6.85, with 95% CI = − 9.56 to − 4.14 and *P*<0.00001 (Fig. 3c). The diamond lattice did not intersect the invalid line and fell to the left of the invalid line. Based on this analysis, the Tb/Sp values of the two groups indicated that BMSCs plus other intervention groups decreased the Tb/Sp value in ovariectomized rats.

## Discussion

The analyses showed that BMD values remarkably increased, indicating that BMSCs accelerated callus maturity and ossification. Moreover, the addition of other therapeutic elements to the BMSCs more dramatically increased healing. Indeed, MSC conditioned media can induce a similar or stronger osteogenic effect than transplanted cells [23]. In the BMSC monotherapy group, the BV/TV (%) value was significantly different, while the Tb/Sp value was not. However, in the BMSCs plus other treatment groups, the results were exactly the opposite: the BV/TV (%) values were not significantly different, whereas the Tb/Sp values were. Overall, the possibility of treating the fracture site in the treatment group was significantly higher than that in the control groups, and the MSC conditioned media for bone regeneration could represent an alternative to cell-based therapies in the future.

BMSCs have the ability to differentiate into osteoblasts, fat, cartilage, neuron, and so on, and have low immunogenicity, and multi-potential differentiation. BMSCs have attracted much attention because of their advantages, such as self-renewal and multidirectional differentiation [24]. Moreover, the advantages of autologous bone marrow mesenchymal stem cell transplantation include no rejection and few post-transplant complications, it can effectively avoid bone defect and healing delay of traditional autologous bone transplantation [25]. Although the classical Ex vivo expanded stem cells has been validated, this requiring a lot of cultivate time before implantation [26, 27]. Several researchers have confirmed that recruitment of endogenous MSC is a viable alternative to MSC transplantation [27–29]. In some studies, BMCS has limited differentiation ability, such as Balakumaran [30] studies have shown that Telomere Biology Disorders (TBD)-BMSCs exhibited reduced clonogenicity, spontaneous differentiation into adipocytes and fibrotic cells, and increased senescence in vitro. Upon in vivo transplantation into mice, TBD-BMSCs failed to form bone or support hematopoiesis, unlike normal BMSCs. Additionally, it has been reported that unmodified MSCs showed oncogenic transformation when injected into immune-compromised mice [31]. But recent studies indicates that reports of oncogenic transformation or malignant of MSCs may reflect the role of cell culture cross-contamination rather than true oncogenic transformation [32].

It is possible that the BMSCs can directly differentiate into osteoblasts in a physiological environment. However, the cytokines from transplanted cells are more likely to play an important role in bone metabolism [33]. Given that BMSC number from marrow decline with age [34]. Whereas, in the treatment of bone diseases, not only BMSCs but also ADSCs [35], muscle-derived stem cells [36], and so on, all can be induced to be divided into osteoblasts. In addition, in clinical aspects, some studies have shown that intracoronary injection of autologous BMSC can improvement in left ventricular function in patients with anterior ST-segment elevation myocardial infarction [37]. Carlos [38] et al., also reported that BMSC were transplanted into the perilesional area in five patients bearing sequels of stroke, with excellent tolerance and without complications. It has also been reported that autologous BMSCs transplantation for the treatment of breast cancer related lymphedema is effective and feasible [39]. Gunter [40] et al., found that in patients with malignant liver lesions, the combination of CD133 BMSC with portal vein embolization administration significantly increased hepatic regeneration. Women have been using stem cell technology for cosmetic indications for the past few years, and there seems to be a reason to believe that stem cells can now be used to solve more serious clinical symptoms [41].

Modern medical treatment of osteoporotic fracture is based on inhibiting bone resorption, promoting bone formation, and regulating blood calcium and blood phosphorus levels to improve pain symptoms [42]. Commonly used drugs are calcium, bisphosphonate, calcitonin, parathyroid hormone, and so on. Moreover, the drugs are combined with nonsteroidal painkillers and physical therapy [43]. Antiresorptive agents fail to adequately restore bone mass and bone quality, and daily injections of parathyroid hormone (PTH) can increase bone mass to stimulate bone formation. However, chronically elevated PTH levels cause bone resorption exceeding bone formation, ultimately resulting in osteoporosis [44] and the risk of developing osteosarcoma. Therefore, treatment options for promoting bone regeneration and reversing bone loss are currently limited. Unfortunately, researchers do not always get the results of clinical trials, and most of the results have not been published in peer-reviewed journals [45]. In short, due to the unsatisfied treatment effect, we investigate the feasibility of BMSCs to provide more clinical treatment in future.

This meta-analysis included various fracture sites (femur, tibia, and mandibles), different initiation times of treatment (1–12 weeks), different BMSC doses and sources, and various measurement standards and calculation methods. Animal information was not comprehensive, such as intervention methods, and outcome indicators, which could cause a high heterogeneity [46]. Thus, random-effects models were used for the analysis and more models of different types of fracture, unified measurement, and same treatment time are needed to verify the efficiency of BMSCs.

### Potential clinical value

BMSCs have great potential for the treatment of osteoporotic fractures in clinical applications. Although a large number of studies have been conducted since the first implantation of stem cells for bone formation or bone regeneration more than 50 years ago [47], so far only a few have been used in clinical practice. According to the updated guidelines of the American College of Physicians, there are limited pharmacologic therapeutic methods to reduce the risk of osteoporosis are reduced [48]. Therefore, it is necessary to study more effective interventions for osteoporotic fractures. This study summarizes the basic research to demonstrate the therapeutic effects of BMSCs on osteoporotic fractures by promoting callus ossification, accelerating callus formation, and strengthening the healed bone. Once BMSCs are proven to be clinically effective, BMSCs have a shorter treatment time and better results. In the future, BMSCs can be used as osteoporotic fracture drugs. Although the applicable type and effective dose have not yet been identified, more rigorous animal model experiments will address this issue before clinical application.

## Conclusions

In summary, as stem cell therapy may develop into a new treatment for osteoporotic fractures, more systematic studies are needed to investigate the regime, safety, and efficacy for fracture healing. Nowadays, the application of BMSCs in the field of orthopedics is developing rapidly. Cell-based strategies have appeared as hopeful treatment strategies when the intrinsic regenerative potential is reduced by health, age, or other factors [8]. The BMSCs could remarkably increased BMD values of osteoporotic fractures Rats. This indicating that BMSCs accelerated callus maturity, promoted ossification of the fracture site and restore the mechanical properties of the bone. Therefore, the role of BMSCs in the treatment of orthopedic diseases has become more important. Although all the research is still in the basic stage, in view of the increasing research in this field, it is believed that BMSC transplantation has a very good clinical application prospect in treating areas of bone and cartilage in the future.

## Data Availability

All data and materials are contained within the manuscript.

## Abbreviations

ADSC: Adipose-Derived Mesenchymal Stem Cells
BMD: Bone mineral density
BMSCs: Bone Marrow Mesenchymal Stem Cells
CI`: Confidence interval
FDA: Food and drug administration
OVX: Ovariectomy
SMD: Standardized mean difference
